# Regulation of apoptosis-inducing factor-mediated, cisplatin-induced apoptosis by Akt

**DOI:** 10.1038/sj.bjc.6604223

**Published:** 2008-02-19

**Authors:** X Yang, M Fraser, M R Abedini, T Bai, B K Tsang

**Affiliations:** 1 Reproductive Biology Unit and Division of Gynaecologic Oncology, Department of Obstetrics & Gynaecology, University of Ottawa, Ottawa, Ontario, Canada K1H 8L6; 2 Department of Cellular & Molecular Medicine, University of Ottawa, Ottawa, Ontario, Canada K1H 8M5; 3 Chronic Disease Program, Ottawa Health Research Institute, Civic Campus, Ottawa, Ontario K1Y 4E9, Canada; 4 Beijing Obstetrics and Gynecology Hospital, Capital Medical University, Beijing 100026, China

**Keywords:** Akt, apoptosis, cisplatin, chemoresistance, AIF

## Abstract

Cisplatin is a first-line chemotherapeutic for ovarian cancer, although chemoresistance limits treatment success. Apoptosis, an important determinant of cisplatin sensitivity, occurs via caspase-dependent and -independent mechanisms. Activation of the protein kinase Akt, commonly observed in ovarian tumours, confers resistance to ovarian cancer cells via inhibition of caspase-dependent apoptosis. However, the effect of Akt on cisplatin-induced, caspase-independent apoptosis remains unclear. We show that in chemosensitive ovarian cancer cells, cisplatin induces the mitochondrial release and nuclear translocation of apoptosis-inducing factor (AIF), a mediator of caspase-independent apoptosis, and AIF-dependent apoptosis. Cisplatin failed to induce these effects in the chemoresistant variant cells. Overexpression of AIF sensitised resistant cells to cisplatin-induced apoptosis. Finally, activation of Akt attenuated the cisplatin-induced mitochondrial release and nuclear accumulation of AIF and apoptosis in chemosensitive cells, whereas inhibition of Akt activity facilitated these effects and sensitised chemoresistant cells to AIF-dependent, cisplatin-induced apoptosis. These results suggest that cisplatin-induced apoptosis proceeds, in part, via a caspase-independent mechanism involving AIF, and that Akt activation confers resistance to cisplatin-induced apoptosis by blocking this pathway. These results provide insights into the molecular mechanism of chemoresistance, and suggest that inhibition of Akt activity may represent a novel therapeutic approach to the treatment of cisplatin-resistant ovarian cancer.

Chemoresistance is a significant barrier to the successful treatment of human ovarian cancer. While many factors affect the sensitivity of cancer cells to chemotherapy, the regulation of a number of key mediators of apoptosis is frequently altered in chemoresistant cells (Asselin *et al*, 2001; Fraser *et al*, 2003; Dan *et al*, 2004).

Apoptosis-inducing factor (AIF) is a mitochondrial intermembrane flavoprotein that is released from the mitochondria and translocates to the nucleus in response to specific death signals (Susin *et al*, 1999; Daugas *et al*, 2000). Nuclear AIF causes large-scale DNA fragmentation and chromatin condensation in a caspase-independent manner (Cregan *et al*, 2002), and recent studies have shown that mitochondrial AIF release is regulated by p53 and the Bcl-2 family (Cregan *et al*, 2002; MacFarlane *et al*, 2002; Kandasamy *et al*, 2003; Majewski *et al*, 2004; Vyas *et al*, 2004).

Akt (protein kinase B) mediates the cell survival action of growth factors and cytokines in a variety of cell types and blocks apoptosis induced by multiple apoptotic stimuli. It is a major downstream target of phosphatidylinositol 3-OH-kinase (PI3K) (Cheng *et al*, 2002; Franke *et al*, 2003; Fresno Vara *et al*, 2004). Both PI3K and Akt are frequently activated and/or overexpressed in human ovarian cancer (Fraser *et al*, 2003; Dan *et al*, 2004). Akt activation promotes cell survival, suppresses apoptotic death and regulates *cis*-platinum (II) diammine dichloride (CDDP) sensitivity in human ovarian cancer cells (Asselin *et al*, 2001; Fraser *et al*, 2003; Dan *et al*, 2004).

We previously demonstrated that activated Akt is an important regulator of both X-linked inhibitor of apoptosis protein and p53 levels after cisplatin (CDDP) challenge and that p53 mutational status is a determinant of Akt-mediated chemoresistance (Fraser *et al*, 2003; Dan *et al*, 2004). Recent data from our laboratory have shown that p53 triggers the Akt-sensitive release of mitochondrial Smac into the cytosol and induces apoptosis via a caspase-dependent pathway (Yang *et al*, 2006). However, other groups have reported that mitochondrial AIF translocation to the nucleus induces caspase-independent apoptosis (Cregan *et al*, 2002). Whether this process contributes to CDDP-induced apoptosis and is regulated by Akt remain unclear.

In the current study, we demonstrate that CDDP-induced mitochondrial AIF translocation to nucleus is a determinant of chemosensitivity in ovarian cancer cells. Decreased mitochondrial AIF release is one mechanism by which Akt-mediated chemoresistance occurs. Our results suggest that modulation of these key cell fate regulators may be an effective means of overcoming chemoresistance in human ovarian cancer.

## MATERIALS AND METHODS

### Reagents

Cells were cultured at 37°C with 5% CO_2_ in RPMI (Roswell Park Memorial Institute, Buffalo, NY, USA)-1640 (OV2008 and C13^*^) or DMEM (Dulbecco's modified Eagle's medium)/F-12 (A2780s, A2780cp; Invitrogen Inc., Burlington, ON, Canada). Medium was supplemented with 10% fetal bovine serum, streptomycin (100 *μ*g ml^−1^), penicillin (100 U ml^−1^), and fungizone (0.625 *μ*g ml^−1^). Digitonin, CDDP, dimethyl sulphoxide, and Hoechst 33258 were supplied by Sigma (Oakville, ON, Canada). Apoptosis-inducing factor small inhibitory RNA was from Santa Cruz Biotechnology (cat. no. sc-29193; Santa Cruz, CA, USA). Negative control siRNA was from Dharmacon Inc. (Chicago, IL, USA). Ribojuice siRNA transfection reagent was from Novagen (San Diego, CA, USA). Adenoviral AIF cDNA and LacZ cDNA were synthesised at the Neuroscience Research Institute, University of Ottawa (Ottawa, ON, Canada). All adenovirus stock solutions were CsCl purified. Primary antibodies were anti-AIF rabbit polyclonal IgG (Santa Cruz Biotechnology), anti-actin mouse monoclonal IgG (Cedarlane Laboratories Ltd, Burlington, ON, Canada), anti-Cox-4 mouse monoclonal IgG (Molecular Probes, Burlington, ON, Canada), and anti-C23 mouse monoclonal IgG (Santa Cruz Biotechnology). Horseradish peroxidase-conjugated anti-mouse and anti-rabbit antibodies for western blot were obtained from Bio-Rad (Hercules, CA, USA). Enhanced chemiluminescent reagents were from Amersham Biosciences (Buckinghamshire, UK).

### Cell culture

Chemosensitive cells (OV2008 and A2780s) and their resistant variants (C13^*^ and A2780cp, respectively) were cultured and treated as reported previously (Fraser *et al*, 2003). Cells were cultured in RPMI-1640 or DMEM/F12, respectively, containing G418 (250 *μ*g ml^−1^; Invitrogen). All CDDP treatments were performed in serum-free media.

C13^*^ cells were stably transfected with pcDNA3.1-DN-Akt2 and A2780s cells were stably transfected with pcDNA3-Myr-Akt2 in the laboratory of Dr Jin Cheng (Moffatt Cancer Center, Tampa, FL, USA) and maintained in RPMI-1640 or DMEM/F12, respectively, each containing G418 (250 *μ*g ml^−1^; Invitrogen).

### Adenovirus infection

Cells were infected with AIF adenoviral constructs as indicated in the text. Infection with LacZ adenovirus was used to normalise the total concentration of adenovirus in each treatment group. Adenovirus infection efficiency was determined as reported previously (Fraser *et al*, 2003).

### Preparation of whole-cell lysates and subcellular fractions

Before fractionation, the cells were cultured and treated as above. Following treatment, cells were washed with ice-cold phosphate-buffered saline, left on ice for 10 min, and then resuspended in homogenisation buffer (250 mM sucrose, 10 mM KCl, 1.5 mM MgCl_2_, 1 mM Na-EDTA, 1 mM Na-EGTA, 1 mM dithiothreitol, 0.1 mM phenylmethylsulphonyl fluoride, and 10 mM Tris-HCl (pH 7.4)) containing the proteinase inhibitor aprotinin (1 *μ*g *μ*l^−1^). After 60 strokes in a Dounce homogeniser, the unbroken cells were spun down at 30 **g** for 5 min. The nuclear and heavy mitochondrial fractions were collected at 750 **g** for 10 min and at 14 000 **g** for 20 min, respectively, from the supernatant. The nuclear fraction was washed three times with the homogenisation buffer containing 0.01% NP-40.

### Western blot analyses

Western blotting was performed as described previously (Fraser *et al*, 2003). Membranes were incubated overnight at 4°C in primary antibodies (anti-AIF, 1 : 500; anti-actin, 1 : 2000; anti-Cox-4, 1 : 1000; anti-LDH, 1 : 1000), followed by incubation with horseradish peroxidase-conjugated anti-rabbit or anti-mouse secondary antibody (1 : 2000) at room temperature for 1 h. Peroxidase activity was visualised with the enhanced chemiluminescence kit. Results were scanned and analysed using Scion Image software (Scion Inc.).

### Assessment of apoptosis

After treatment, cells were harvested and the percentage of apoptosis was determined by Hoechst 33258 staining (12.5 ng ml^−1^), as reported previously (Abedini *et al*, 2004). Cells were counted with the counter ‘blinded’ to sample identity to avoid experimental bias.

### Statistical analyses

Results are presented as mean±s.e.m. of at least three independent experiments. Data were analysed by two-way ANOVA and Bonferroni post-test to test the differences between groups (PRISM software version 3.0; GraphPad, San Diego, CA, USA). Statistical significance was inferred at *P*<0.05.

## RESULTS

### CDDP induces nuclear translocation of mitochondrial AIF and apoptosis in chemosensitive, but not chemoresistant, ovarian cancer cells

**Figure 1:**
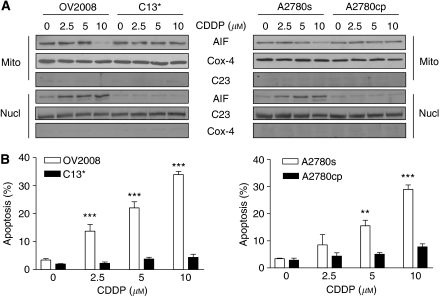
CDDP induced mitochondrial AIF translocation to nucleus and apoptosis in chemosensitive, but not resistant, ovarian cancer cells. Chemosensitive (OV2008, A2780s) and resistant (C13^*^, A2780cp) cells were treated with different concentrations of CDDP (0, 2.5, 5, and 10 *μ*M) for 24 h. CDDP decreased mitochondrial and increased nuclear AIF contents (**A**) as well as induced apoptosis (**B**) in a concentration-dependent manner in OV2008 and A2780s cells, but not C13^*^ and A2780cp cells (^**^P<0.01, ^***^P<0.001). Cox-4 blots indicate mitochondrial loading. C23 was used as a loading control for the nuclear fraction. The reciprocal blots demonstrate purity of the preparations. CDDP-induced apoptosis was assessed by Hoechst staining.

To determine the relationship between subcellular AIF distribution and CDDP sensitivity, chemosensitive ovarian cancer cells (OV2008 and A2780s) and their resistant variants (C13^*^ and A2780cp, respectively) were cultured with different concentrations of CDDP (24 h; 0, 2.5, 5, and 10 *μ*M; dimethyl sulphoxide as control). Mitochondrial and nuclear fractions were prepared and AIF levels were determined by western blot. C23 and Cox-IV were used as purity controls for mitochondria and nucleus, respectively. As shown in Figure 1A, CDDP decreased mitochondrial and increased nuclear AIF contents in OV2008 and A2780s cells but not in C13^*^ or A2780cp cells. Furthermore, the accumulation of AIF in the nucleus of chemosensitive cells was associated with an increase in the percentage of cells undergoing apoptosis, as measured by evaluation of nuclear morphology by Hoechst 33258 nuclear staining. However, CDDP failed to induce apoptosis in C13^*^ or A2780cp cells during CDDP treatment (Figure 1B).

**Figure 2:**
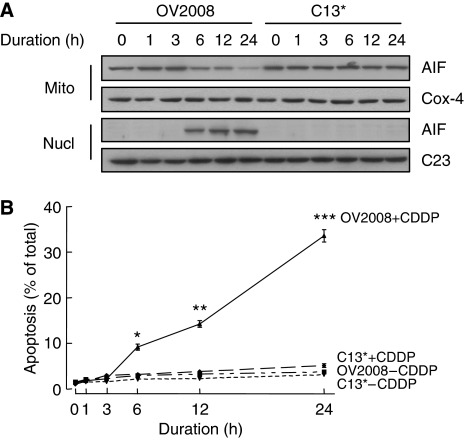
CDDP induced mitochondrial AIF translocation (**A**) and apoptosis (**B**) in a time-dependent manner in chemosensitive, but not resistant, ovarian cancer cells. OV2008 and C13^*^ cells were treated with 10 *μ*M CDDP for different durations (0–24 h). CDDP induced decrease in mitochondrial and increase in nuclear AIF contents in OV2008 cells in a time-dependent manner, but not in C13^*^ cells (^*^P<0.05, ^**^P<0.01, ^***^P<0.001).

In addition, we extended these concentration–response experiments to include time-course studies. OV2008 and C13^*^ cells were treated with 10 *μ*M CDDP for different durations (0–24 h). It was observed that CDDP decreased mitochondrial AIF levels and induced apoptosis in the chemosensitive ovarian cancer cells (OV2008), but not in the resistant variant (C13^*^). These changes were time-dependent (Figure 2). Taken together, these data demonstrate that CDDP induces mitochondrial AIF translocation and apoptosis in chemosensitive, but not chemoresistant, ovarian cancer cells.

### AIF is required for CDDP-induced apoptosis

**Figure 3:**
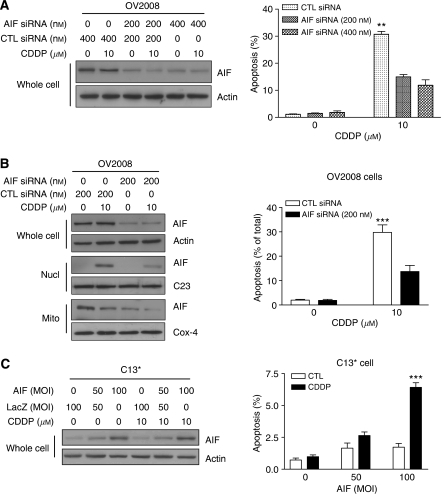
Apoptosis-inducing factor is required for CDDP-induced apoptosis in ovarian cancer cells. (**A**) OV2008 cells were transfected with AIF siRNA (0–400 nM; 48 h) and then treated with CDDP (10 *μ*M; 24 h). Downregulation of AIF was confirmed by western blot. Downregulation of AIF significantly attenuated CDDP-induced apoptosis in OV2008 cells (^**^P<0.01). (**B**) OV2008 cells were tranfected with 200 nM AIF siRNA for 48 h, followed by CDDP treatment (10 *μ*M; 24 h). Transfection of 200 nM AIF siRNA decreased AIF content in whole cell and mitochondria and decreased AIF translocation to nucleus. Downregulation of AIF decreased CDDP-induced apoptosis in OV2008 cells (^***^P<0.001). (**C**) C13^*^ cells were infected with adenoviral AIF for 48 h, followed by CDDP treatment (10 *μ*M; 24 h; ^***^P<0.001).

To determine whether AIF is required for CDDP-induced apoptosis, OV2008 cells were transfected with AIF or control siRNA (0–400 nM) for 48 h and then treated with 10 *μ*M CDDP for 24 h. Downregulation of AIF was confirmed by western blot (Figure 3A and B). Apoptosis-inducing factor siRNA (200–400 nM) markedly decreased whole-cell AIF content, and these changes significantly attenuated CDDP-induced apoptosis in these cells (^**^P<0.01; Figure 3A). We extended this experiment to a single concentration of AIF siRNA (200 nM) and subcellular AIF levels were measured by western blot. As shown in Figure 3B, 200 nM AIF siRNA significantly attenuated whole-cell AIF level as well as mitochondrial and nuclear AIF content. The lower AIF content was associated with decreased CDDP-induced apoptosis in OV2008 cells (^***^P<0.001).

We next determined whether an increased AIF level is sufficient to sensitise resistant cells to CDDP-induced apoptosis. C13^*^ cells were infected with adenoviral AIF (MOI (multiplicity of infection)=0, 50, and 100; LacZ control) for 48 h, followed by incubation of CDDP (10 *μ*M) for 24 h. Apoptosis-inducing factor markedly sensitised the resistant cells to CDDP-induced apoptosis in a concentration-dependent manner (^***^P<0.001; Figure 3C). Taken together, these results demonstrate that AIF is an important mediator of CDDP-induced apoptosis and that overexpression of AIF is an effective means of overcoming chemoresistance in ovarian cancer cells.

### Akt inhibits CDDP-induced mitochondrial AIF translocation to nucleus in ovarian cancer cells

**Figure 4:**
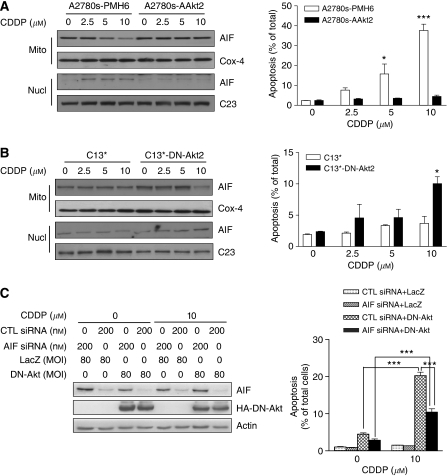
Akt regulates CDDP-induced mitochondrial AIF translocation and apoptosis in ovarian cancer cells. (**A**) Activated Akt2 inhibited mitochondrial AIF translocation and CDDP-induced apoptosis. A2780s-PMH6 (control) and A2780s-AAkt2 (active Akt2) cells were treated with CDDP (0–10 *μ*M; 24 h). Compared with A2780s-PMH6 cells, A2780s-AAkt2 cells showed a significant suppression of CDDP-induced AIF translocation. Constitutively activated Akt2 also reduced the sensitivity of A2780s cells to cisplatin-induced apoptosis (^*^P<0.05, ^***^P<0.001) expressing DN-Akt2 in response to CDDP. (**B**) Inhibition of Akt2 sensitised C13^*^ cells to CDDP (^*^P<0.05). (**C**) C13^*^ cells were infected with DN-Akt (MOI=80; LacZ control) in the presence or absence of AIF siRNA (200 nM). DN-Akt sensitised the cells to CDDP-induced apoptosis, which was attenuated by downregulation of AIF (^***^P<0.001).

Activation of Akt impairs the cellular stress response and confers resistance to CDDP-induced apoptosis in tumour cells (Dan *et al*, 2004). Moreover, we previously demonstrated that Akt suppresses apoptotic cell death and regulates CDDP sensitivity in ovarian cancer cells (Asselin *et al*, 2001; Fraser *et al*, 2003). However, if and how Akt regulates CDDP-induced nuclear translocation of mitochondrial AIF is unknown, as is the involvement of this process in the regulation of chemosensitivity. To determine if Akt regulates CDDP-induced AIF translocation, chemosensitive ovarian cancer cells (A2780s) stably transfected with constitutively active Akt2 (myristoylated wild-type Akt2; A2780s-AAkt2) were cultured with different concentrations of CDDP (0, 2.5, 5, and 10 *μ*M). In control-transfected cells (A2780s-PMH6), CDDP decreased mitochondrial AIF level and increased nuclear AIF contents, whereas these responses were absent in A2780s-AAkt2 cells, suggesting that Akt activation suppresses CDDP-induced nuclear translocation of mitochondrial AIF (Figure 4A).

To further ascertain the role of Akt2 in the regulation of mitochondrial AIF translocation in CDDP-induced apoptosis in ovarian cancer cells, we extended these observations with a concentration–response (0, 2.5, 5, and 10 *μ*M) study using the chemoresistant ovarian cancer cell line C13^*^ and C13^*^-DNAkt2 cells, which are C13^*^ cells stably transfected with dominant-negative Akt2 (T308A, S473A). Western blotting analyses demonstrated that downregulation of Akt2 facilitated a CDDP-induced decrease in mitochondrial AIF content and an increase in nuclear AIF level, and sensitised chemoresistant ovarian cancer cells to CDDP-induced apoptosis (Figure 4B). These findings suggest that Akt2 regulates CDDP-induced AIF translocation and is a determinant of chemoresistance in ovarian cancer cells.

To confirm that AIF is involved in Akt-mediated chemoresistance, we infected chemoresistant C13^*^ cells with adenoviral HA-DN-Akt (MOI=80; LacZ control) in the presence or absence of control or AIF siRNA (200 nM). As shown in Figure 4C, AIF siRNA markedly downregulated AIF content. Moreover, while expression of DN-Akt sensitised the cells to CDDP-induced apoptosis (*P*<0.001), downregulation of AIF significantly attenuated this response, suggesting that AIF is downstream of Akt and that Akt-mediated chemoresistance involves, in part, the disruption of AIF function.

## DISCUSSION

Mitochondrial membrane permeabilisation is considered to be one of the initial events of the apoptotic process induced by chemotherapeutic drugs. Opening of the mitochondrial permeability transition pore, which is under the control of members of the Bcl-2 family, results in the permeabilisation of the outer mitochondrial membrane and subsequent release of apoptogenic proteins such as cytochrome *c* and Smac (Kuwana *et al*, 2002; Green, 2005). In the present study, we have demonstrated that CDDP induced mitochondrial AIF translocation in chemosensitive ovarian cancer cells, but not in their resistant variants, in a time- and concentration-dependent manner, suggesting that AIF translocation may be a determinant of CDDP-induced apoptosis. Downregulation of AIF by siRNA conferred resistance to chemosensitive cells, suggesting that AIF release is required for CDDP-induced apoptosis. While there is evidence that AIF release is induced by a wide range of cellular insults, including chemotherapeutic agents (Alfano *et al*, 2006; Massard *et al*, 2006), our data represent, to our knowledge, the first report directly demonstrating that mitochondrial AIF translocation to nucleus is an important determinant of CDDP-induced apoptosis.

CDDP-based chemotherapy is an important treatment modality, and the development of chemoresistance is a major hurdle limiting successful treatment. However, the molecular mechanisms underlying chemoresistance are poorly understood. It is well established that CDDP induces cell death by activating death receptor- and mitochondria-mediated apoptosis (Alfano *et al*, 2006; Massard *et al*, 2006). Caspases are a family of cysteine proteases that have been implicated as key effector molecules in the execution of apoptotic cell death (Cryns and Yuan, 1998). Many of the characteristic morphological and biochemical changes that occur during apoptosis result from activation of caspases. More than 10 caspases have been identified. While some (e.g., caspase 8 and 10) are involved in the initiation of apoptosis, others (e.g., caspase 3, 6, and 7) execute cell death by destroying essential proteins.

Although caspases are important mediators of apoptosis, there is accumulating evidence indicating the existence of caspase-independent mechanisms of CDDP-induced cell apoptosis (Barton *et al*, 2005). It has been reported that AIF mediates cell death through a caspase-independent pathway (Cregan *et al*, 2002). Mitochondrial AIF translocates to the nucleus following a death stimulus and initiates nuclear condensation. Once the nucleus condenses, this leads to large-scale chromatin fragmentation, followed by cell death. In the present study, we found that AIF nuclear translocation is also required for CDDP-induced apoptosis, suggesting that apoptotic cell death induced by CDDP may also be AIF-dependent. While these results are in agreement with those of Henkels and Turchi (1999), they also represent, to our knowledge, the first demonstration that AIF is required for CDDP-induced apoptosis, and suggest that AIF may contribute to the observed caspase-independent apoptosis induced by CDDP.

Akt is a major downstream effector of PI3K (Franke *et al*, 1995). Akt promotes cell survival, suppresses apoptosis, and regulates CDDP sensitivity in ovarian cancer cells (Asselin *et al*, 2001; Fraser *et al*, 2003; Dan *et al*, 2004). In a previous study, we demonstrated that Akt regulates Smac release and apoptosis by attenuating the mitochondrial actions of p53 (Yang *et al*, 2006). Our present data also show that mitochondrial AIF translocation to nucleus is regulated by Akt during CDDP-induced apoptosis in ovarian cancer, suggesting that activation of Akt promotes chemoresistance, in part, by attenuating CDDP-induced AIF translocation from mitochondria to nucleus. This result also suggests that Akt may inhibit both caspase-dependent and -independent apoptosis signalling pathways, thus providing a potential explanation for the potent anti-apoptotic effects of this molecule.

The mechanisms by which Akt may regulate AIF nuclear translocation are not clear. Apoptosis-inducing factor does not contain a consensus Akt phosphorylation site. As such, it is unlikely that the observed effects of Akt on AIF are due to a direct interaction between the molecules. Alternatively, Akt may act at the mitochondria, directly attenuating the release of pro-apoptotic proteins from the mitochondria to the cytoplasm. Akt is known to phosphorylate and inactivate proteins, such as Bad, implicated in mitochondrial pore formation, so it is possible that this plays a significant role in mediating the anti-apoptotic effects of Akt. Finally, we have previously demonstrated that Akt attenuates translocation of p53 to the mitochondria (Yang *et al*, 2006), and that mitochondrial p53 can directly stimulate the release of pro-apoptotic proteins from the mitochondria. As such, it is possible that the inhibition of nuclear AIF accumulation induced by Akt activation is secondary to the inhibition of p53-dependent release of AIF from the mitochondria. The precise mechanism underlying this phenomenon is currently under investigation in our laboratory.

In summary, we report here that translocation of AIF from mitochondria to the nucleus is disrupted in chemoresistant cells, relative to their sensitive counterparts, and that AIF is required for CDDP-induced apoptosis. Furthermore, we have demonstrated that failure to induce mitochondrial release and nuclear accumulation of AIF may be an important determinant of resistance to CDDP in ovarian cancer cells, and that Akt activation may confer resistance, in part, through modulation of this pathway.
